# COVID-19 Vaccine Clinical Trials: A Bird’s Eye Perspective

**DOI:** 10.7759/cureus.28066

**Published:** 2022-08-16

**Authors:** Pujitha Vallivedu Chennakesavulu, Gaurav Venkat Cuddapah, Mayura Reddy Keesara, Jyothik Varun Inampudi, Amulya Arremsetty, Sushma Moka, Swamy Miryala

**Affiliations:** 1 Internal Medicine, Kamineni Academy of Medical Sciences and Research Centre, Hyderabad, IND; 2 Internal Medicine, Apollo Institute of Medical Sciences and Research, Hyderabad, IND; 3 Internal Medicine, Siddhartha Medical College, Vijayawada, IND

**Keywords:** severe acute respiratory syndrome coronavirus-2 (sars-cov-2), sars-cov-2, covid-2019, infectious and tropical diseases, preventive health, covid 19, clinical trials in all phases (i-iv), covid-19 vaccine

## Abstract

Several Phase-III clinical studies investigating vaccine safety and effectiveness have been published a year following the first breakout of the COVID-19 pandemic. These vaccine candidates were produced using a variety of vaccination technologies, including mRNA, recombinant protein, adenoviral vector, and inactivated virus-based platforms, by various research organizations and pharmaceutical firms. Despite many successful clinical studies, participants are restricted by trial inclusion and exclusion criteria, geographic location, and the current state of the virus epidemic. Many concerns remain, particularly for specific populations such as the elderly, women who are pregnant or nursing, and teenagers. Vaccine effectiveness against asymptomatic infection and particular viral variations, on the other hand, is still largely unclear. This review will focus on vaccination candidates that have completed Phase-III clinical trials and will examine the scientific evidence that has been gathered so far for these vaccine candidates for various subgroups of individuals and virus variations.

## Introduction and background

The SARS-CoV-2 pandemic is a worldwide crisis that is yet to be addressed. There are more than 585 million verified COVID-19 cases worldwide, and this illness has taken the lives of more than 6.4 million people. More than 12.3 billion vaccine doses have been administered with 5.3 billion persons vaccinated with at least one dose and 4.8 billion persons fully vaccinated. The adoption of stringent measures in certain nations or cities, including extensive testing, isolation of affected people, and lockdown of a portion of the country or area, along with an uncontrollable increase in the number of cases, may have halted the epidemic. Nevertheless, it is fraught with social difficulties, psychological difficulties, and economic difficulties. Not only are COVID-19 patients and front-line healthcare professionals at an augmented danger of mental health ailments that have a noteworthy impact on their everyday lives, but there is a reduction in the optimistic emotions and life satisfaction among the general inhabitants too, as well as the elderly people, which may have an impact on their mental health and increase their risk of developing mental ailments [1].

The competition to manufacture a SARS-CoV-2 vaccine has resulted in vaccine candidates based on a range of approaches, including both traditional methods, such as live-attenuated viruses, inactivated whole viruses, and virus protein subunits, and next-generation techniques, such as DNA, messenger RNA (mRNA), and viral vector-based technologies. According to WHO and FDA recommendations, the minimum requirement for an approved COVID-19 vaccine is clear evidence of at least 50% vaccination effectiveness. The WHO recommended assessing illness, severe disease, and/or transmission as trial endpoints, whereas the FDA recommended laboratory-confirmed COVID-19 or SARS-CoV-2 infections as key endpoints for vaccination effectiveness [2].

The COVID-19 vaccine trials initially started with 64 vaccine candidates against SARS-CoV-2, which were in clinical stages of development a year after the epidemic began. Various vaccines are presently undergoing Phase-III clinical trials, the findings of which have been published in peer-reviewed publications or publicized in press releases. Two mRNA-based vaccination systems, two viral vector-based vaccine platforms, and one protein subunit-based vaccine platform are among them. Four of these have already received clearance in the United States and Europe like Pfizer-Biotech, Moderna, Janssen and Novavax. Thanks to advancements in vaccine technology, such as mRNA vaccines that can be effortlessly customized to different infections, vaccine research has accelerated to unprecedented levels, never seen before COVID-19. Other vaccine candidates have been authorized outside of the United States and Europe, in addition to these three. Some of them are now prepared to satisfy the demands of various health authorities and will be released soon [3].

Researchers have outlined what characteristics make an excellent SARS-CoV-2 vaccine candidate for fighting the pandemic in a review article, though none of the candidates accomplish all of the requirements. Furthermore, as we learn more about SARS-CoV-2 during the ongoing pandemic, new criteria have emerged, implying that, despite prevailing success in Phase-III clinical trial programs of various vaccine candidates, there are still many unanswered questions that the scientific community must address before we recognize the best vaccine candidates for various conditions [4]. These questions include: First, whether the candidate vaccine elicits a long-term protective immune response and can be demonstrated to be safe with long-term follow-up. Second, whether the vaccine can be vaccinated to people with various conditions, such as the ageing population, adolescents, and concomitant diseases such as pregnant or lactating women. Third, whether vaccines can elicit an immune response that protects people from certain diseases.

This study will go through published preclinical and clinical data on different vaccines that have been in trials. In this study, we also report the established efficacy in determining over various comorbidities and adverse effects of vaccines.

## Review

Methods

Data Source and Strategy

“Few specific vaccines which are in phase 3 trials” OR Covid (“SARs-CoV-2” OR pandemic) OR ”corona virus” OR “vaccine trials” OR “vaccine candidates” OR ”vaccine administration” OR immunity OR “chimpanzee adenoviral vector” OR “spike protein*” OR “mice and rhesus macaques” OR “humoral and cell-mediated response” OR “first dose” OR “second dose” OR “two doses” OR “neutralizing antibody” OR vaccination OR “vaccine efficacy” OR “safety profile” OR “adverse event*” OR “limitation*” OR “follow-up” OR “intramuscular route” OR BioNtech OR Pfizer OR “nucleoside modified RNA” OR “local response” OR “systemic reactions” “first covid-19 vaccination for emergency use” OR Moderna OR ”SARS-CoV-2 glycoprotein with a transmembrane anchor” OR “National Institute of Allergy and Infectious Disease” OR “anti-SARS-CoV-2 immune responses” OR “unintentional adverse events” OR “offer long-term humoral immunity” OR Janssen OR “recombinant replication-incompetent adenovirus” OR Novavax* OR “Matrix-M1 adjuvant” OR “wild-type spike glycoprotein” OR “CoronaVac” OR “Sinovac inactivated viral vaccine” OR “double-blind placebo-controlled clinical study”.

Study Selection and Eligibility Criteria

Authors have collected extensive data after identifying relevant papers. After screening titles and abstracts, full-text articles were evaluated for eligibility. On relevant full-text articles, a quality check was performed. Only publications that passed a 70% quality rating check were included in the study. We looked for types of vaccines and their mechanisms of action and their effects in different age and ethnic groups that were published in English. The response of our bodies to vaccines is included in the study. The data has been collected in different phases of the vaccination trials and their effectiveness is mentioned. The relevant adverse effects were identified and mentioned. Along with the overall efficacies of the vaccines are included. COVID-19 and SARS-CoV-2 are used in line with the nomenclature used in the relevant research.

Review

ChAdOx1 nCoV-19 Adenoviral Vector Vaccine

A replication-deficient chimpanzee adenoviral vector carrying the SARS-CoV-2 spike protein gene was created at Oxford University and is known as ChAdOx1 nCoV-19 (AstraZeneca). In mice and rhesus macaques, it has been found to cause a strong humoral and cell-mediated response. Rhesus macaques were likewise protected from SARS-CoV-2-induced pneumonia when they were vaccinated with ChAdOx1 nCoV-19. The first dose of this vaccine was shown to produce antigen-specific antibody and T-cell responses in animal models, whereas the second dose was shown to enhance antibody responses and increase SARS-CoV-2 neutralizing antibody [5]. In a Phase-I/II trial, two doses of the vaccine were found to increase anti-spike protein neutralizing antibody titers, Fc-mediated functional antibody responses, antibody-dependent neutrophil/monocyte phagocytosis, complement activation, and natural killer cell activation, indicating that two doses should be used in future clinical trials. Another Phase-I/II research found that this vaccine has a favourable safety profile and can elicit antibody responses in the majority of people. Similarly, the Phase-II/III research revealed that this vaccine is well tolerated and capable of eliciting immunogenicity in the majority of trial participants. Voysey et al. conducted a four-clinical-study interim review of the effectiveness and safety of the ChAdOx1 nCoV-19 vaccine during the trials and they found it to be 70% efficacious against COVID-19 [6]. The vaccination showed an excellent safety profile, with the number of severe adverse events and adverse events of particular concern being evenly distributed throughout the trial arm. The most common adverse effects reported were muscle aches, fever and headache [7]. No life-threatening adverse events were reported according to randomized control trials by Alghamdi et al. [8].

BNT162b2 mRNA Vaccine

Prior to the COVID-19 pandemic, no mRNA medication or vaccine has been approved for human use. BioNTech and Pfizer collaborated on BNT162b2. It is a full-length spike protein encoded by a lipid nanoparticle-formulated nucleoside-modified RNA. Among the four possible mRNA vaccine candidates, this vaccine candidate was chosen for future clinical development based on evidence of its capacity to elicit neutralizing antibodies with a reduced frequency and severity of systemic responses [9]. A study by Polack et al. reported that Phase-II/III trials had resulted in vaccine effectiveness of over 95% [10]. The local reactions were mild to moderate in intensity and dissipated in one to two days, with no participant reporting a grade 4 local response after the two injection doses. Systemic reactions, such as fever and chills, appeared one to two days after immunization and disappeared quickly. Treatment-related adverse events were reported by 21% more individuals in the vaccination arm (21%) than in the control arm (5%), with this increase owing mostly to the inclusion of transitory occurrences. Only four vaccine-related severe adverse events were recorded in the vaccination arm, and the investigators found no fatalities linked to the vaccine or placebo. Along with these common side effects, myocarditis cases were found on rising after the second dose [11]. 

Post-vaccination cardiac adverse events have been reported by the vaccine adverse event reporting system (VAERS) database with the highest risk of atrial fibrillation (AF) [12]. Among the vaccines, BNT162b2 has been found to have the highest incidence of AF. Anaphylaxis was found in a small sample of the population receiving the BNT162b2 vaccine [13]. Based on the findings, the USFDA approved BNT162b2 as the first COVID-19 vaccination for emergency use [14].

mRNA-1273 mRNA Vaccine

Moderna mRNA-1273 encodes the S-2P antigen, which consists of the SARS-CoV-2 glycoprotein with a transmembrane anchor and an intact S1-S2 cleavage site, which was co-developed by the National Institute of Allergy and Infectious Disease. The robustness of mRNA-1273 in generating SARS-CoV-2 neutralizing action, protecting the lower airways and upper airways as well as the lung, was shown in animal research [15]. mRNA-1273 was able to elicit anti-SARS-CoV-2 immune responses in all subjects in Phase-I research, with no trial-limiting safety concerns. The vaccine's safety profile was also evaluated in older people, with the majority of adverse events being mild or moderate in the phase 1 trial. Although these reactions decreased somewhat over time, they remained high in all 34 healthy adult individuals assessed 90 days following the second immunization. These findings indicate that mRNA-1273 may offer long-term humoral immunity as per phase 1 trials [16]. Despite this, mRNA-1273 exhibited a high degree of binding and neutralizing antibody responses in a Phase-I study, according to an update of the immunogenicity data [17]. There were 30,420 individuals in the Phase-III trial, with 185 instances of COVID-19 detected in the control arm and 11 cases in the vaccination arm, resulting in vaccine effectiveness of 94.1%. In the vaccination arm, unanticipated adverse events at the injection site were more common than in the control arm. Nonetheless, they were mostly of grade 1 or 2 intensity and lasted just two to three days on average after the first or second dosage. Solicited systemic adverse responses were more common in the vaccination arm than in the control arm, and the severity of the systemic events increased in the second dosage compared to the first dose, although the effects persisted only two to three days on average after the first or second doses. The incidence of unintentional adverse events, severe adverse events, and serious adverse events recorded within the 28 days after injection, on the other hand, was almost the same in the vaccination and control groups. Cardiac adverse events like pericarditis, AF and myocarditis were reported much more often in patients with second doses of vaccination as reported by the VAERS database [12].

Ad26.COV2.S Adenovirus Vector Vaccine

Janssen is the company behind Ad26.COV2.S. It is a full-length SARS-CoV-2 spike protein encoded by a recombinant replication-incompetent adenovirus serotype 26 vector. The vaccine was shown to be safe in the Phase-I research, with just five out of 401 individuals reporting severe side events, and no one dropping out due to an adverse event. Furthermore, neutralizing antibodies were found in all individuals, indicating that Ad26.COV2.S immunization was highly immunogenic. The findings of the Phase-I study, taken together, justify the vaccine's inclusion in a Phase-III trial. A preliminary review of 468 symptomatic COVID-19 cases revealed that this single-dose vaccine had a vaccine effectiveness of 66% in avoiding moderate and severe COVID-19 at 28 days post-vaccination, according to the Phase-III study's 44,325 participants [18]. Thrombosis with thrombocytopenia syndrome (TTS), a rare syndrome with thrombosis of arteries and veins and thrombocytopenia, and Guillain Barre Syndrome (GBS) were confirmed adverse events by committees after the single dose vaccination in April 2021 [11]. Thus, the benefits and the efficacy of the single-dose vaccine outweigh the risks associated with the vaccine.

NVX-CoV2373 Protein Subunit Vaccine

Novavax's NVAX-CoV2373 is a protein subunit vaccine that includes Matrix-M1 adjuvant as well as a recombinant SARS-CoV-2 full-length wild-type spike glycoprotein. The vaccine has been evaluated in a variety of animal models, and it has been shown to induce immunogenicity and offer protection against SARS-CoV-2 infection in these animals [19]. The vaccine was shown to be safe and generated adequate immune responses in a Phase-I trial. A Phase-III trial in the United Kingdom found overall vaccination effectiveness of 89.3%, including 86% against the newly discovered variation B.1.351 [20]. The two-dose regimen of the NVX-Co2373 has shown the efficacy of 96.3% against beta variant and 86.3% against alpha variant in a phase III study by Heath et al. [21]. In a study by Keech et al., NVX-CoV2373 appeared to be safe, and it elicited immune responses that exceeded levels in COVID-19 convalescent serum [22].

*CoronaVac Inactivated Virus Vaccine* 

CoronaVac is a Sinovac inactivated viral vaccine that induces an immune response against multiple antigens of the SARS-CoV-2 virus rather than only the spike protein. The effectiveness and safety of this inactivated viral vaccine were evaluated by healthcare professionals in a Phase-III double-blind placebo-controlled clinical study that used a two-dose intramuscular injection schedule with a 14-day gap [23]. A Phase-II data indicated that seroconversion of neutralizing antibodies was more than 97% with an adverse response rate of less than 35%.

The firm also revealed the results of its Phase-III study, which showed vaccination effectiveness of 50.7% for all cases, 84% for patients needing medical care, and 100% for severe, fetal cases and cases requiring hospitalization [24]. Pityriasis rosea and Reactive arthritis are a few of the rare adverse events reported post the vaccination along with the common injection site pain, headache and fatigue [25].

Vaccine efficiency and protection in diverse populations

Most Phase-III clinical trials evaluate vaccination effectiveness and safety in a relatively limited human population and under extremely specific circumstances, such as restricting participants to a certain age range, testing in just a few countries, and excluding individuals who are pregnant or have other problems. As a consequence, it may be difficult to extrapolate Phase-III findings to10 a larger population or to certain circumstances. As a result, a slew of follow-up studies has looked into particular human groups or settings to see whether the findings can still be trusted in these situations. The elderly have been the worst hit by the COVID-19 pandemic, not only because they are more vulnerable to SARS-CoV-2 infection, but also because they have more severe life-threatening COVID-19 symptoms [26]. Due to the deterioration of their immune system, comorbidities, and pharmaceutical therapies, their immunological response to vaccination may vary from that of the younger population.

The majority (88%) of the patients in the ChAdOx1 nCoV-19 Phase-III study were between the ages of 18 and 55, according to the main efficacy analysis. The vaccine's effectiveness was consistent across age subgroups in the BNT162b2 Phase-III trial. Participants aged 16 years to 55 years old had 95.6% vaccination effectiveness, while those aged 55 years, 65 years, and 75 years old had 93.7%, 94.7%, and 100% vaccine efficacy, respectively [6].

The vaccination effectiveness for individuals aged 18 to 65 in the mRNA-1273 Phase-III trial was 95.6% for those aged 18 to 65, and 86.4% for those aged 65 and above. A Phase-I dose-escalation trial of mRNA-1273 was extended to include 40 older individuals, 20 of whom were between the ages of 56 and 70, and another 20 beyond the age of 70. Only one person missed the second dosage owing to the development of a maculopapular rash, which investigators determined was unrelated to immunization. The binding and neutralizing antibody responses seemed to be comparable in these older adult participants in this expansion trial compared to those aged 18 to 55 years, and the vaccination evoked a robust CD4 cytokine response in this older group [27].

More clinical data focused on this group is needed to completely explain the benefit-risk ratio to inform healthcare providers and educate the public throughout the immunization campaign, particularly in regions with significant vaccine reluctance. Paediatricians and teenage populations are another unreached age group in all of these COVID-19 vaccination Phase-III clinical trial investigations. Lack of evidence in these groups will also lead to vaccination reluctance, particularly among parents of young children who have milder signs of COVID-19 infection due to SARS-CoV-2 infection. More evidence from pediatric and adolescent populations will be critical in developing SARS-CoV-2 herd immunity. COVID-19 may have varied effects on individuals of various ethnicities, according to some evidence. The bulk of participants in published Phase-III COVID-19 trials was white, with extremely low participation percentages among other ethnic groups, including black populations. The majority (83%) of the individuals included in the main effectiveness analysis in the ChAdOx1 nCoV-19 Phase-III study were white, although the findings in other ethnic groups were not addressed. The vaccination effectiveness for white individuals in the BNT162b2 trial was 95.2%, whereas it was 93.9% for people of other ethnicities [10]. The vaccination effectiveness for white people in the mRNA-1273 trial was 93.2%, whereas it was 97.5% for non-white people [28].

The evidence on vaccine effectiveness and safety for people with comorbidities and particular diseases has been in recent studies There was the highest incidence and death from diabetes post-vaccination of Ad26.COV2.S as compared to other vaccines [12]. The vaccination effectiveness in obese individuals was reported in the BNT162b2 trial. The results showed that vaccination effectiveness was comparable in the subgroups of individuals with obesity (95.4%) and those without obesity (94.8%). Even when age and obesity were combined, vaccine efficacy has remained over 90% in all categories [29]. The vaccination can also prevent those who are at risk of severe COVID-19, according to the mRNA-1273 Phase-III study. According to the emergency use authorization fact sheet, the only known information about the COVID-19 vaccine and pregnancy comes from a reproductive toxicity study in female rats, which assessed vaccine-related adverse effects on female fertility, fetal development, and postnatal development with no adverse events reported. For data gathering, a pregnant exposure registry has been established to track the result of women who have been vaccinated with mRNA-1273 [30].

Vaccine effectiveness in defending the growth of stern COVID-19 

The vaccination effectiveness for asymptomatic infection was extremely low in the ChAdOx1 nCoV-19 vaccine Phase-III trial, with seventy-one cases identified among the 5000+ participants, thirty-seven in the control arm, and thirty-four in the vaccine arm, yielding a vaccine efficiency of just 7.8%. According to these findings, ChAdOx1 nCoV-19 may not be the best vaccine option for preventing SARS-CoV-2 transmission in the asymptomatic stage [31]. Ten COVID-19 patients required hospitalization in the same study; all were from the control arm, and two of them developed severe COVID-19, including one fatal case, suggesting that ChAdOx1 nCoV-19 is effective in avoiding severe COVID-19. The BNT162b2 research did not look at asymptomatic infection. In terms of severe COVID-19 protection, nine out of 20,000+ people in the control group developed severe COVID-19, while one person in the vaccine group had severe COVID-19, resulting in an 89% efficacy. There were 30 individuals in the mRNA-1273 Phase-III study who developed severe COVID-19, and all of them were in the control group, showing vaccination effectiveness against severe COVID-19 development of cent percent [32]. These findings indicate that, although it's unclear if these vaccine candidates can stop SARS-CoV-2 from spreading, they may significantly reduce SARS-potential CoV-2 to cause severe symptoms when infected.

Vaccine efficacy and COVID-19 variants

With the current augmentation of case numbers in South Africa and the United Kingdom, as well as the discovery of novel variants in these 2 nations, the question of whether the tested vaccine candidates can protect us from these variants remains unanswered, raising public concerns about their efficacy in the authentic scenario. In the UK, these new variations are known as B.1.1.7, whereas in South Africa, they are known as B.1.351 [33]. The D614G mutation is present in the UK variation B.1.1.7, along with eight additional spike protein alterations. The D614G mutation is found in the South African variation B.1.351, along with nine additional spike protein alterations [34]. These two variations have been shown in vitro to be resistant to neutralization by a monoclonal antibody against the spike protein and the receptor-binding domain to varying degrees, raising questions about vaccination effectiveness against these recently emerging variants.

Sera from 40 people who were vaccinated with BNT162b2 was able to neutralize both the B.1.1.7 lineage and the Wuhan reference strain that has recently appeared in the UK, notwithstanding to an inferior degree, demonstrating that BNT162b2 can still draw defense against the B.1.1.7 lineage [33]. Similarly, 20 human sera from the BNT162b2 study had analogous neutralizing titers to the Y501 viruses and N501 viruses, representing that BNT162b2 may offer defense against the novel viral strain that takes place from South Africa and UK. The potential of the mRNA-1273 vaccination to induce neutralizing antibodies against various spike mutations from SARS-CoV-2 variants has also been studied recently. Six SARS-CoV-2 variations were examined in the research, and none had a significant effect on neutralization against the B.1.1.7 form that arose in the United Kingdom. Despite this, there was a lower level of neutralization against the mutations seen in the B.1.351 variety that emerged from South Africa. Despite the lower neutralization reaction, vaccinated individuals' sera still had a high degree of neutralization capacity [35].

Another research found that plasma from a group of 20 volunteers who received either the mRNA-1273 or the BNT162b2 vaccinations acquired plasma neutralizing activity. However, activity against SARS-CoV-2 variants encoding E484K or N501Y, or the K417N: E484K: N501Y combination, was significantly lowered, suggesting the possibility of clinical vaccination effectiveness is lost. This may happen when the spike protein of SARS-CoV-2 accumulates mutations over time, implying that mRNA vaccines may need to be updated on a regular basis to prevent clinical effectiveness loss. Although the findings of the NVX-CoV2373 vaccine's Phase-III data have yet to be published in a peer-reviewed publication, early data released in a press release indicated that this vaccine had 86% vaccination effectiveness against the variant strain emerging from the UK. Furthermore, the Phase-IIb trial of this vaccine was conducted as the South African 501Y.V2 escape mutant was discovered to be dominating the illness in South Africa, and the findings revealed that the total vaccination effectiveness was 49.4%. The 501Y.V2 escape variant from South Africa was found in 93% of SARS-CoV-2 infected people, according to sequencing data. These findings suggest that NVX-CoV2373 can protect against the South African SARS-CoV-2 variant, albeit to a lower degree than the original and UK strains [36]. Various Phase-III COVID-19 Vaccines are depicted in Table 1.

**Table 1:** Different phase-III COVID-19 vaccines Vaccine efficacies and safeties in different populations and against different SARS-CoV-2 variants.

Vaccine	Age group	Efficacy	Efficacy with ethnicity	Efficacy with comorbidities
Ad26. COV2.S	18+	85%	NR	NR
BNT162b2	16-55	95.6%	95.2% for Caucasian 93.9% for Other than Caucasian	95.4% with obesity 94.8% without obesity
ChAdOx1 nCoV-19	18-55	70.4%	83% for Caucasian	NR
CoronaVac	18+	50.7%	NR	NR
mRNA-1273	18-65	95.6%	93.2% for Caucasian, 97.5% for Other than Caucasian	NR
NVX-CoV2373	18-65	89.3%	NR	NR

## Conclusions

The first-generation approval of the COVID-19 vaccines is just the first step in fighting SARS-CoV-2. Although the vaccine efficacy represented is affected by various existing variants. The review article proposes that the vaccines in these phase III clinical trials have good neutralizing antibodies against the SARS-CoV-2, thus decreasing the incidence. The general complications of vaccines included injection site pain, headaches, and myalgias but rare adverse events are reported by COVID-19 vaccines. Severe cardiac events (pericarditis and myocarditis) are substantially reported by mRNA vaccines like BNT162b2 mRNA vaccine and mRNA-1273 mRNA vaccine. GBS and TTS are oftentimes associated with Ad26.COV2.S adenoviral vector vaccine. Despite these adverse events the risk-benefit analysis suggests that vaccination surpasses the contrary. The current prevailing studies are established on two doses of vaccines but not the booster dose of vaccines. Thus, more studies are required to check the long-term efficacy of these vaccines to the emerging variants along with long-term adverse events.
